# GC Preps: Fast and Easy Extraction of Stable Yeast Genomic DNA

**DOI:** 10.1038/srep26863

**Published:** 2016-05-31

**Authors:** Benjamin A. Blount, Maureen R. M. Driessen, Tom Ellis

**Affiliations:** 1 Centre for Synthetic Biology and Innovation, Imperial College London, London, United Kingdom; 2 Department of Bioengineering, Imperial College London, London, United Kingdom

## Abstract

Existing yeast genomic DNA extraction methods are not ideally suited to extensive screening of colonies by PCR, due to being too lengthy, too laborious or yielding poor quality DNA and inconsistent results. We developed the GC prep method as a solution to this problem. Yeast cells from colonies or liquid cultures are lysed by vortex mixing with glass beads and then boiled in the presence of a metal chelating resin. In around 12 minutes, multiple samples can be processed to extract high yields of genomic DNA. These preparations perform as effectively in PCR screening as DNA purified by organic solvent methods, are stable for up to 1 year at room temperature and can be used as the template for PCR amplification of fragments of at least 8 kb.

The isolation of genomic DNA (gDNA) from *Saccharomyces cerevisiae* can be laborious and time-consuming, particularly during extensive screening procedures in which many samples are prepared. As it becomes possible to assemble progressively larger synthetic DNA sequences, typified by the construction of whole synthetic yeast chromosomes, or make multiple parallel changes to genomic loci, such as with CRISPR-mediated genome editing techniques, screening requirements also scale up as more gDNA samples need to be analysed by more polymerase chain reactions (PCRs)^1^,^2^,^3^. For the example of synthetic chromosome assembly, each *in vivo* construction stage requires the preparation of gDNA from many colonies and dozens of PCRs to be carried out on each of these samples to differentiate between synthetic and natural amplification target sites^4^.

Previously, researchers facing these challenges have used a mixture of colony boil PCRs, which are fast and easy but yield a low quality product, and phenol-chloroform-isoamyl alcohol (PCI) DNA extraction methods, which yield higher quality gDNA but are more time and labour intensive and use hazardous chemicals^1^. Other gDNA extraction methods tend to be lengthy, such as those with long zymolyase incubations to disrupt the cell wall, are prohibitively expensive for intensive use or rely on purification steps involving organic solvents^5^,^6^,^7^,^8^,^9^. Methods such as lithium acetate-sodium dodecyl sulphate (LiOAc-SDS) extraction, which was developed as a rapid way of extracting gDNA for PCR-based applications, are faster than traditional PCI extractions but still involve an ethanol precipitation stage and are difficult to scale up when processing many samples in parallel^10^. A method of gDNA extraction is required that yields DNA that can reliably be used as a PCR template but is also fast, cheap and easy to perform. Here, we describe a gDNA extraction method, the glass bead Chelex 100 preparation (GC prep), that meets these requirements.

## Results and Discussion

### GC prep development

In developing a gDNA extraction method, vortex mixing cells with glass beads was selected as an effective way of lysing yeast cells, as demonstrated in previous methods^6^. Purification of crude lysates is often the most time-consuming part of gDNA extraction, but pure DNA is not necessary for most PCR-based applications. By boiling the lysate with Chelex 100 resin, cellular components are denatured and the Chelex 100 collates polyvalent metal ions, thereby reducing the activity of any nucleases which require them as co-factors^11^. These steps are intended to increase DNA preparation stability within the crude lysate.

A GC prep gDNA extraction was initially performed on pelleted cells from 100 μl of saturated overnight culture of *S. cerevisiae* BY4742. Cells were resuspended in 100 μl of a suspension of 5% Chelex 100 and glass beads were added. The samples were vortex mixed for 4 minutes then incubated at 100 °C for 10 minutes before centrifugation for 1 minute, with the supernatant being used as the preparation. This method yielded gDNA that was successfully amplified by PCR. Resuspending a single colony, rather than pelleted culture, in 100 μl 5% Chelex 100 and then following the same protocol also yielded gDNA that could be PCR amplified.

**Figure 1:**
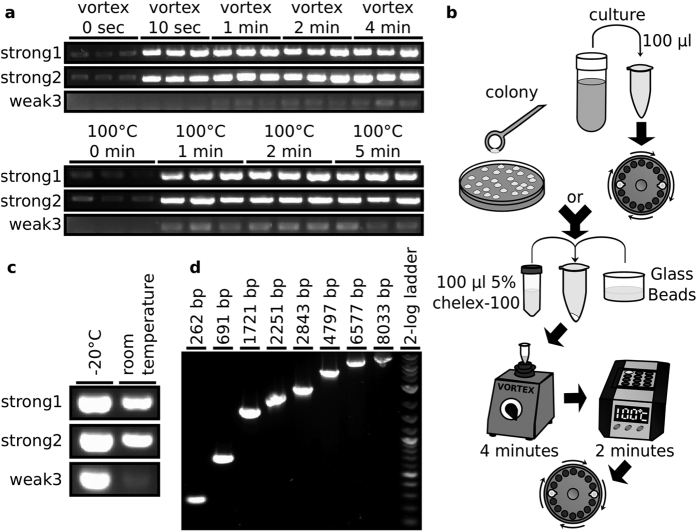
Optimisation and evaluation of the GC prep method. (**a**) PCR amplifications performed on GC preps with varied vortex and boil times in triplicate. Where vortex time was varied, 100 °C incubation time was 2 minutes and where 100 °C incubation time was varied, vortex time was 4 minutes. (**b**) Schematic diagram of the optimised GC prep method. (**c**) PCR amplifications performed on GC preps that had been incubated for 1 year at either −20 °C or room temperature. (**d**) PCR amplification of progressively larger amplicons using a GC prep as template.

To optimise the GC prep, extractions with a range of vortex and boiling times were performed in triplicate on saturated culture. To determine which variations produced gDNA best suited to PCR applications, the preparations were amplified with three pairs of PCR primers (Fig. 1a). All primers were targeted to amplify regions in the coding sequence of the YKL068W gene, with two primer pairs, YKL068W_1_WT_F/ YKL068W_1_WT_R and YKL068W_2_WT_F/ YKL068W_2_WT_R, typically producing strong product bands and one primer pair, YKL068W_3_WT_F/ YKL068W_3_WT_R, typically producing weak product bands when analysed by gel electrophoresis. These primer pairs will be referred to as strong1 (with an amplification product 463 bp in length), strong2 (490 bp) and weak3 (289 bp) respectively. A vortex time of 4 minutes produces the brightest band with the weak3 primers and a 100 °C incubation time of 2 minutes appears to be optimal, producing consistently bright bands. Unless otherwise stated, these were the parameters used for subsequent GC preps. A schematic of the optimised GC prep protocol is shown in Fig. 1b.

### GC preps are stable and effective PCR templates

To assess the stability of the gDNA in the preparations, GC preps produced by a 4 minute vortex and 10 minute boil time were incubated at either room temperature or −20 °C for 1 year before undergoing PCR amplifications (Fig. 1c). The GC preps at −20 °C for 1 year were strongly amplified in each PCR reaction. The GC preps that had been left at room temperature for 1 year also showed strong amplification with the strong1 and strong2 primers and showed amplification, albeit weaker than for the −20 °C preps, with the weak3 primers. As strong PCR product bands can be produced using GC preps that have been left at room temperature for 1 year, it is clear that the Chelex 100 boil step has successfully removed almost all nuclease activity from the preparations and that they are very stable.

The strong1, strong2 and weak3 primer pairs are all used to amplify products less than 500 bp in size. To ensure that larger products can be PCR amplified from GC preps, PCRs were performed with a range of primers to amplify products ranging from 262 bp to 8 kb using a GC prep as the template (Fig. 1d, Supplementary Tables S1 and S2). All of the PCRs successfully amplified the correctly sized product.

### Comparing GC preps to alternative methods

**Figure 2:**
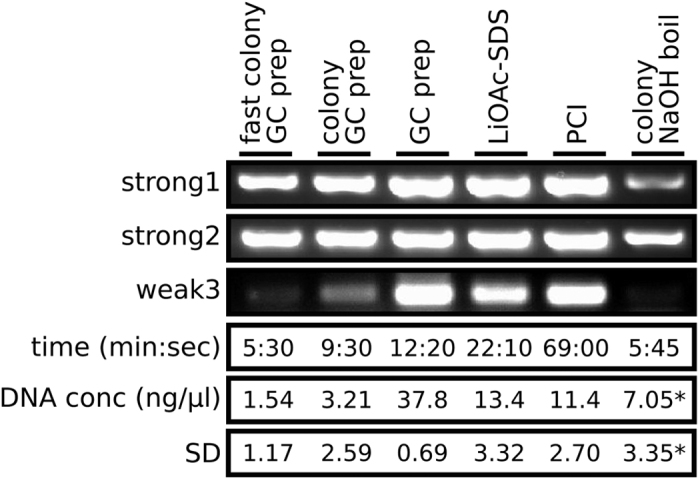
Comparison of the GC prep to other methods. Each preparation was performed in triplicate. Preparations were used as template for PCR amplification, a representative example is shown for each method. The time given is the time taken to generate the three gDNA preparations for each method, from colony or culture to finished preparation. DNA conc is the mean value of the DNA concentrations of each of the triplicate preparations, the asterisk denotes that the colony NaOH values are adjusted for final volume differences when compared to other methods. SD is the standard deviation from the mean of the DNA concentrations of the triplicate preparations.

The GC prep method was developed as an alternative to colony boil and PCI gDNA extraction methods. To increase the flexibility of the method, GC preps using single colonies as the starting material were developed, with colony GC preps using the optimised vortex and boil times, and fast colony GC preps using reduced vortex and boil times of 1 minute each. The performance of GC preps, colony GC preps and fast colony GC preps were compared to colony boil, PCI and LiOAc-SDS methods (Fig. 2). A single BY4742 colony picked from an agar plate was used to inoculate a single 5 ml overnight liquid culture. This was used as the starting material for triplicate GC prep, PCI and LiOAc-SDS preparations. Other single BY4742 colonies from the same plate were used as the starting material for triplicate fast colony GC preps, colony GC preps and NaOH colony boil preparations. For extraction of gDNA from single colonies, the colony GC prep resulted in the brightest PCR gel bands and the fast colony GC prep also showed brighter bands than the NaOH colony boil method whilst taking marginally less time to prepare. The GC prep performed very favourably when compared to the PCi and LiOAc methods, being faster and having a higher and more consistent gDNA concentration. The GC prep showed PCR product bands that were at least as bright as any of the other methods assessed.

### Scaling to 96-well format

To demonstrate that the GC prep method is suitable for 96-well format, 90 colony GC preps were performed on BY4741 colonies within a 96 well plate. To determine whether cross-well contamination occurs at the vortex stage, the remaining six wells were not inoculated with a colony and were processed as a negative control. These samples, along with 4 individual colony GC preps and 4 NaOH colony boil preps, were used as template for PCR amplification using strong1 primers (Supplementary Figure S1). Each of the inoculated samples, and none of the negative control samples, produced a strong1 amplicon band showing that all of the preparations were successful and that there was no observed cross-contamination. There was some variation in band brightness but this does not appear to be due to any effects of well location within the plate.

## Discussion

The GC prep method was developed to be a fast and easy way of extracting gDNA from yeast that is of sufficient quality for in depth PCR screening. GC preps fulfil these criteria and provide an effective PCR amplification template. When compared to alternative gDNA extraction methods, they are faster and perform at least as well as PCR templates (Fig. 2). The method is inexpensive, with the current reagent cost per GC prep being around 0.05 GBP (0.07 USD), as well as scalable to 96-well format. The stability and consistency of the GC preps, along with the ability to use them as a template for PCR amplification of amplicons up to at least 8 kb in length, points to the method being an attractive option whenever yeast gDNA extraction is required for PCR applications.

## Methods

### GC prep protocol

**Reagents -** 5% Chelex 100 (95577, Sigma Aldrich) in deionised water, acid washed glass beads (G8772, Sigma Aldrich).

**Equipment -** 1.5 ml microtubes, vortex genius 3 shaker (IKA) fitted with a universal attachment adaptor (IKA, VG3.3), Dri-Block heater (Techne), microcentrifuge.

**Step 1 -** In a 1.5 ml microtube, resuspend pelleted 100 μl overnight yeast culture in 100 μl 5% Chelex 100.

**Step 2 -** Add glass beads to half total sample volume and vortex mix at high speed for 4 minutes.

**Step 3 -** Incubate sample at 100 °C for 2 minutes.

**Step 4 -** Spin microtube at top speed in a microcentrifuge for 1 minute. The supernatant is the GC prep and can be transferred to a fresh microtube, ensuring that no Chelex 100 is carried over.

### Colony GC prep

Colony GC preps are performed as above, substituting a single colony for the pelleted cells in step 1. Fast colony GC preps have reduced vortex and 100 °C incubation times of 1 minute each.

### 96 well colony GC prep

Glass beads were applied to each well of a 96 well PCR plate (Life Technologies, N8010560) by cutting off the end of a standard 1000 μl pipette tip with a scalpel blade, pouring the beads into the tip, attaching a 1000 μl pipette set to the lowest volume and fully depressing the plunger rapidly three times into each well. To each well, 100 μl 5% Chelex 100 was added and a single yeast colony was suspended in the mix. The plate was sealed with an aluminium plate seal (6524AL, Corning), vortexed at high speed for 4 minutes and incubated at 100 °C for 2 minutes in a thermocycler. The plate was cooled on ice for 10 minutes before carefully removing the plate seal and using 0.2 μl from the top of each sample as template for PCR.

For other gDNA extraction methods used, see Supplementary Information.

### PCR amplifications

PCR amplifications were performed using GoTaq G2 Green Master Mix (M7823, Promega) except for the 262 bp to 8 kb varied length PCRs, which were performed using Phusion High-Fidelity DNA Polymerase (M0530, New England Biolabs). For primer sequences and reaction conditions, see Supplementary Information. PCR products were visualised by 1% agarose gel electrophoresis with SYBR Safe DNA Gel Stain (Thermo Fisher Scientific) and photographed while exposed to ultraviolet light with an exposure time that varied between gels depending on band intensity.

### DNA quantification

Quantification of gDNA concentrations was performed using a Qubit 2.0 Fluorometer (Thermo Fisher Scientific) using a Qubit dsDNA HS Assay Kit (Q32851,Thermo Fisher Scientific). All gDNA preparations assayed had a final volume of 50 μl except for the NaOH colony boil preparations, which had a volume of 5 μl. To account for this, the concentration values for these preparations were adjusted to a tenth of that measured.

### Growth media and conditions

Cells were grown in YPD liquid medium (10 g/l yeast extract, 20 g/l peptone, 20 g/l glucose) or on YPD solid medium (10 g/l yeast extract, 20 g/l peptone, 20 g/l glucose, 20 g/l agar) at 30 °C. Liquid cultures were shaken at 250 rpm.

## Additional Information

**How to cite this article**: Blount, B. A. *et al.* GC Preps: Fast and Easy Extraction of Stable Yeast Genomic DNA. *Sci. Rep.* **6**, 26863; doi: 10.1038/srep26863 (2016).

## Supplementary Material

Supplementary Information
