# Supplementary material for: GC Preps: Fast and Easy Extraction of Stable Yeast Genomic DNA
Source: Sci Rep. 2016 May 31;6:26863. doi: 10.1038/srep26863 (PMC4886510; doi:10.1038/srep26863)
Supplement: Supplementary Information [file srep26863-s1.pdf]

GC Preps: Fast and Easy Extraction of Stable Yeast Genomic DNA

Benjamin A. Blount[1,2], Maureen R.M. Driessen[1,2] and Tom Ellis[1,2,*]

[1]Centre for Synthetic Biology and Innovation, Imperial College London, London, United Kingdom

[2]Department of Bioengineering, Imperial College London, London, United Kingdom

*t.ellis@imperial.ac.uk

Supplementary Information

Supplementary Results

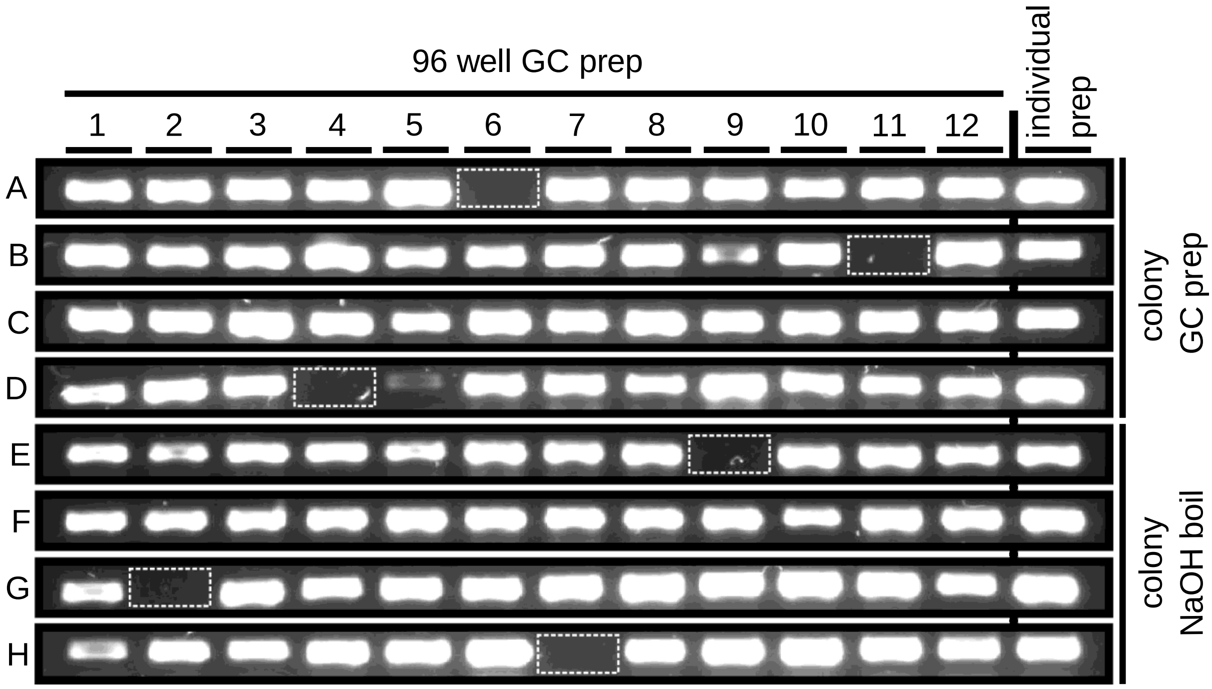

Supplementary Figure S1. Scaling GC preps to 96-well format. Bands shown are strong1 amplicons amplified from 96-well colony GC preps, negative control preps and individually prepared colony GC and colony NaOH boil preps. Well coordinate labels correspond to the plate well the gDNA was prepared in and white dashed boxes denote negative control wells not inoculated with a colony.

Supplementary Methods

Phenol Chloroform Isoamyl alcohol (PCI) preparation

From a saturated 5 ml culture of *S. cerevisiae* BY4742, 100 μl was pelleted by centrifugation in a 1.5 ml microtube for 2 minutes at 4300 rcf and resuspended in 200 μl breaking buffer (50 mM Tris pH8, 100 mM NaCl, 1% SDS, 2% triton X-100, and 1 mM EDTA). To this, a volume of glass beads (G8772, Sigma Aldrich) equal to the resuspended sample and 200 μl PCI (25:24:1) was added and cells were vortexed for 10 minutes then centrifuged at 14,100 rcf for 10 min. From the top aqueous layer, 75 μl was transferred to a fresh microtube containing 1 ml 100% ethanol. The tube was inverted 5 times and centrifuged at 14,100 rcf for 20 minutes at 4 °C. The supernatant was discarded and the DNA pellet was washed with 500 μl 70% ethanol before centrifuging at 14,100 rcf for 5 minutes. The supernatant was removed and the pellet was air-dried before resuspending in 50 μl 10 mM Tris (pH 7.4).

Lithium Acetate Sodium Dodecyl Sulphate (LiOAc-SDS) preparation

From a saturated 5 ml culture of *S. cerevisiae* BY4742, 100 μl was pelleted by centrifugation in a 1.5 ml microtube for 2 minutes at 4300 rcf and resuspended in 100 μl of a solution of 200mM LiOAc and 1% SDS. The sample was vortexed briefly and then incubated at 70 °C for 5 minutes. 300 μl 96% ethanol was added to the sample before briefly vortexing and centrifuging at 14,100 rcf for 3 minutes. The pellet was washed with 500 μl 70% ethanol before centrifuging at 14,100 rcf for 3 minutes. The pellet was resuspend in 100 μl deionised water and then centrifuged at 14,100 rcf for 1 minute. The supernatant was transferred to a fresh tube and used as the DNA preparation.

NaOH colony boil

A single BY4742 colony was suspended in 5 μl 20 mM NaOH and incubated at 100 °C for 5 minutes.

PCR reaction conditions

GoTaq G2 Green 10 μl PCR reactions were made up with 5 μl 2x GoTaq G2 Green Master Mix (M7823, Promega), 0.2 μl gDNA, 1 μl of each primer at 5 μM stock concentration and 2.8 μl deionised water. The touchdown PCR cycle used is shown in Supplementary Fig. S2 and the sequences of primers used are shown in Supplementary Table S1.

Phusion 10 μl PCR reactions were made up with 1 μl HF Buffer (New England Biolabs), 0.2 μl gDNA, 0.2 μl 10 mM dNTPs (New England Biolabs), 0.3 μl DMSO (New England Biolabs), 1 μl of each primer at 5 μM stock concentration, 0.2 μl Phusion polymerase (2000 units/ml, M0530, New England Biolabs) and 6.2 μl deionised water. The Phusion touchdown PCR cycle used is shown in Supplementary Fig. S1. The sequences of primers used are shown in Supplementary Table S1.

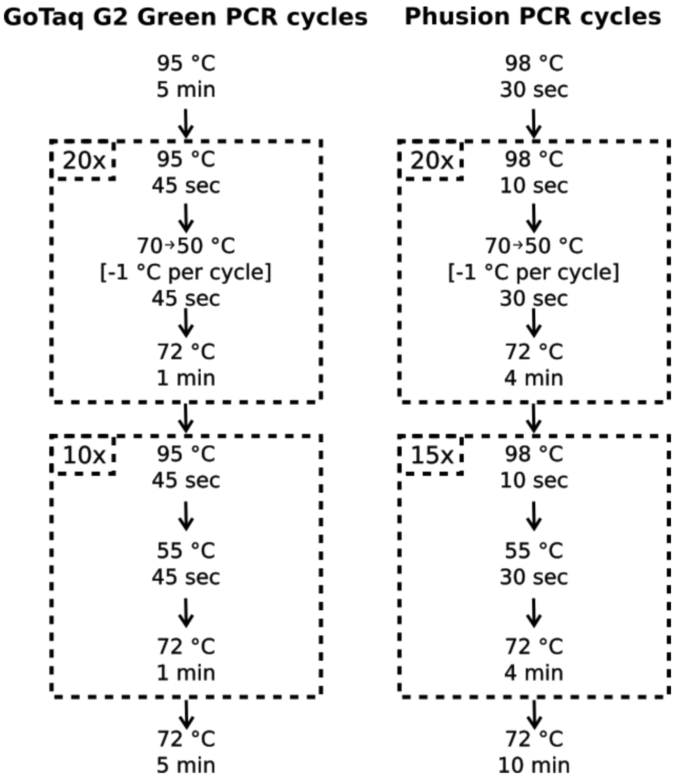

Supplementary Figure S2. PCR cycles used in this study.

Primer Name	Sequence
YKL068W_1_WT_F	CTCACCATTTGGGTCGTTAAACTCCTCA
YKL068W_1_WT_R	ACTGCCCCCCAGCTGATTTTGATTACCA
YKL068W_2_WT_F	CACCTTTTCAAATTCCGCATCAGGAGGT
YKL068W_2_WT_R	GGAAGGTACTGTCGTAGAAGCTGTGTTG
YKL068W_3_WT_F	AGTTGGTTCAGGGTCGCTGTTTGGC
YKL068W_3_WT_R	GGACCCTTGCTGTTGATTATTTTGGCCA
YKL078W_1_WT_F	AGCCAGAAGTGACGTCACATCTCCTGTG
YKL077W_1_WT_F	TACGGTCATTGCCGGTGTCACTTTTTCT
YKL075C_1_WT_F	TCTTTCCAGTCTTTCTTGGCTAACGGCA
YKL074C_2_WT_F	TGATCGACAGGCCAAAACCATAGTGCTA
YKL074C_2_WT_R	TTCTCCGGCTTACTACAACATGGCCTCA
YKL074C_1_WT_R	CGGTGAGCTTCCGAAAGCGCCAAAA
YKL073W_3_WT_R	TGCAACAGACATCCCTTCACTAACCAGA
YKL073W_2_WT_R	AGAGCCTCCCGCCAAAATGACCCCATTT
YKL073W_1_WT_R	ATGCTCATGCAAACGCGAACGTTCTGAA

Supplementary Table S1. Sequences of primers used in this study.

Primer 1	Primer 2	Amplicon Length (bp)
YKL074C_2_WT_F	YKL074C_2_WT_R	262
YKL074C_2_WT_F	YKL074C_1_WT_R	691
YKL074C_2_WT_F	YKL073W_3_WT_R	1721
YKL074C_2_WT_F	YKL073W_2_WT_R	2255
YKL074C_2_WT_F	YKL073W_1_WT_R	2843
YKL075C_1_WT_F	YKL073W_1_WT_R	4797
YKL077W_1_WT_F	YKL073W_1_WT_R	6577
YKL078W_1_WT_F	YKL073W_1_WT_R	8033

Supplementary Table S2. Primer combinations used to produce varied length products by PCR.
